# Regressing eruptive disseminated pigmented Spitz (Reed) nevi in a young adult^☆^^☆☆^

**DOI:** 10.1016/j.abd.2020.11.009

**Published:** 2021-09-24

**Authors:** Belen Lozano-Masdemont, Berta Pérez-Tato, Elena Zamora-Martínez, Enrique Rodríguez-Lomba

**Affiliations:** a Department of Dermatology, Hospital Universitario de Móstoles, Madrid, Spain; b Department of Dermatology, Hospital General Universitario Gregorio Marañón, Madrid, Spain

**Keywords:** Dermoscopy, Melanoma, Nevus

## Abstract

Eruptive disseminated Spitz nevi is a rare clinical presentation that features an abrupt widespread eruption of Spitz nevi. Spontaneous regression of these nevi has been rarely reported in previous literature. The authors of the present study report the case of a 30-year-old man who presented eruptive disseminated Spitz nevi that appeared within a week and started regression in the following years.

## Case report

**Figure 1:**
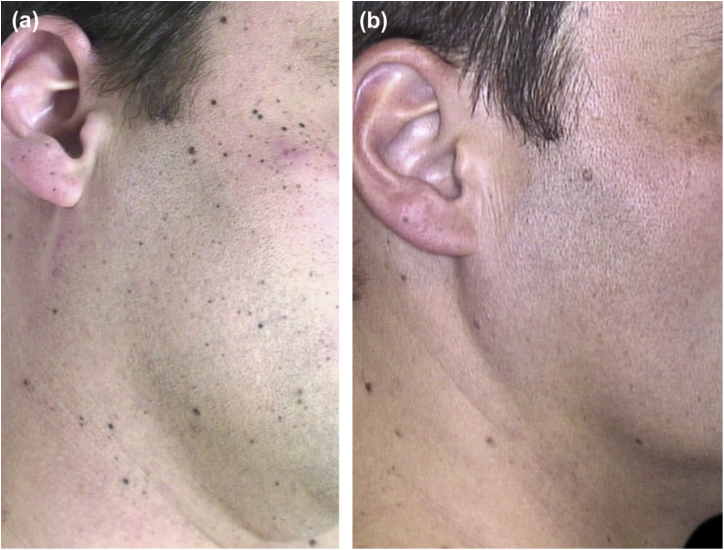
Clinical follow-up images. (A), Multiple small black macules on the face and neck. (B), 7 years later most of the Spitz nevi have completely regressed.

**Figure 2:**
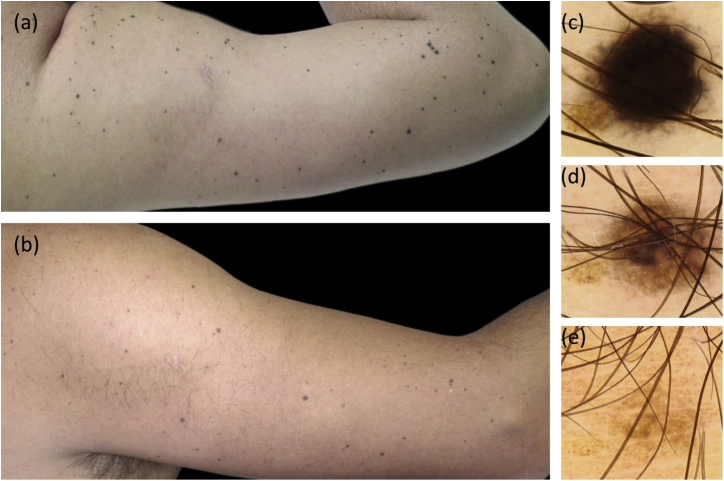
Clinical and dermoscopic follow-up images. (A), Multiple small black and brown macules on the right arm. (B), 7 years later most of the Spitz nevi have completely regressed. (C–E) Dermoscopic follow-up of a representative nevus. C, Starburst pattern: central area of homogeneous black pigmentation and symmetrically distributed peripheral streaks. D, Brown homogeneous pattern. E, Regressing nevus.

A 30-year-old Caucasian healthy man presented a one-month history of hundreds of asymptomatic small black lesions that had appeared within a week, scattered all over the upper half of the body. At this time, he was suffering from work stress. He had Fitzpatrick skin type III, brown hair, and eyes, and had suffered few sunburns during his childhood. There was no family history of dysplastic nevi or melanoma. On physical examination, a multitude of dome-shaped brown-black papules and macules, ranging in size from less than 1 mm to 4 mm, were evident on the head, neck, trunk, and upper limbs (Fig. 1A and Fig. 2A). On dermoscopy, the so-called starburst pattern was predominantly observed, consisting of a central area of homogeneous black pigmentation and regular peripheral streaks, while other lesions presented a homogeneous pattern. Histologic examination of two lesions, taken in two consecutive years, revealed a proliferation of pigmented spindle-shaped melanocytes arranged in junctional nests, confirming the presumptive diagnosis of eruptive disseminated pigmented Spitz (Reed) nevi. Over the next 3 years, the rate of new nevi diminished. Five years after the onset, melanocytic lesions became paler and over the next two years, most of them showed complete regression (Fig. 1B and Fig. 2B-E). Histologic examination of a regressed nevus revealed residual hyperpigmentation with a sparse superficial perivascular lymphocytic infiltrate and melanophages.

## Discussion

Eruptive disseminated Spitz nevi (EDSN) is a rare clinical presentation consisting of an abrupt widespread eruption of Spitz nevi. The pathogenesis is unknown although some possible precipitating factors have been described including perioperative stress, sun exposure, pregnancy, intravenous drug abuse, chemotherapy, and Addison disease.^1^, ^2^, ^3^, ^4^, ^5^, ^6^

EDSN most frequently appears on the trunk, buttocks, and proximal limbs, and usually occurs in the second to third decade of life.^1^, ^2^, ^3^, ^4^, ^5^, ^6^, ^7^, ^8^, ^9^ To the best of the authors’ knowledge, less than 30 cases of EDSN have been published to date.^1^, ^2^, ^3^, ^4^, ^5^, ^6^, ^7^, ^8^, ^9^ EDSN generally exhibits an abrupt, eruptive onset over few months, followed by a slowly progressive course of few lesions that continue to appear over months to several years, resulting in hundreds of lesions. In all reported cases, the EDSN lesions involved the trunk, often affecting the legs, arms, and sometimes the scalp.^6^ In 33% of the cases, the color of the nevi was homogeneously pink; 33% brownish pink; 15% dark brown-black; and the rest were polymorphous.^6^ Most cases with long-term follow-up (almost 20 years) have not shown involution. In four cases spontaneous regression has been documented, as in the present case.^1^, ^7^, ^8^

Specific genetic mutations in solitary Spitz nevi have not been identified in multiple lesions examined from the same patient.^9^ Unfortunately, the authors have not been able to perform a genetic analysis on the samples from the present case.

The differential diagnosis of pigmented EDSP includes agminated eruptive melanocytic nevi (clusters of Spitz nevi in a segmental distribution), dysplastic nevus syndrome, or metastatic melanoma.

Despite the hundreds of Spitz nevi in the same patient, no malignant transformation has been reported.^2^, ^3^, ^4^, ^5^, ^6^, ^7^, ^8^, ^9^ Surgical treatment is not feasible in EDSN due to the high number of lesions and, as no malignant transformation has been reported, long-term follow-up by clinical and dermoscopic photography is a reasonable management option. Ricci et al. have proposed total body photography and dermoscopic follow-up every 3–6 months during the eruptive phase (extending to 9–12 months during the stable phase).^6^ Prompt surgical excision of lesions that become suspicious is recommended.

In summary, EDSN is a rare and impressive clinical presentation of Spitz nevi whose pathogenesis remains unknown. The authors consider that dermatologists should be aware that this entity has shown a good prognosis in reported cases, despite the hundreds of lesions.

## Financial support

None declared.

## Authors’ contributions

Belen Lozano-Masdemont: Approval of the final version of the manuscript; critical literature review; data collection, analysis, and interpretation; effective participation in research orientation; intellectual participation in propaedeutic and/or therapeutic management of studied cases; manuscript critical review; preparation and writing of the manuscript; study conception and planning.

Berta Pérez-Tato: Approval of the final version of the manuscript; effective participation in research orientation; intellectual participation in propaedeutic and/or therapeutic management of studied cases; manuscript critical review; preparation and writing of the manuscript; study conception and planning.

Elena Zamora-Martínez: Approval of the final version of the manuscript; effective participation in research orientation; intellectual participation in propaedeutic and/or therapeutic management of studied cases; manuscript critical review.

Enrique Rodríguez-Lomba: Approval of the final version of the manuscript; critical literature review; intellectual participation in propaedeutic and/or therapeutic management of studied cases; manuscript critical review; preparation and writing of the manuscript; study conception and planning.

## Conflicts of interest

None declared.
